# Computational methods in physical virology: a critical perspective across lengths and timescales

**DOI:** 10.1093/femsre/fuag050

**Published:** 2026-09-22

**Authors:** Adolfo B Poma, Luis F Cofas-Vargas, Fernando L Barroso da Silva, Sergio Pantano, Marta Bally

**Affiliations:** Department of Biosystems and Soft Matter, Institute of Fundamental Technological Research, Polish Academy of Sciences, ul. Pawińskiego 5B, 02-106 Warsaw, Poland; Departamento de Química, Universidad Autónoma Metropolitana-Iztapalapa, 09310 Mexico City C.P., Mexico; Department of Biomolecular Sciences, School of Pharmaceutical Sciences at Ribeirão Preto, University of São Paulo, 14040-900 Ribeirão Preto, São Paulo, Brazil; Department of Chemical and Biomolecular Engineering, North Carolina State University, 27695 Raleigh, North Carolina, United States; Institut Pasteur de Montevideo, Mataojo 2020, 11400 Montevideo, Uruguay; Área Bioinformática, Departamento DETEMA, Facultad de Química, Universidad de la República, 11800 Montevideo C.P., Uruguay; Department of Clinical Microbiology, Wallenberg Centre for Molecular Medicine, Umeå Centre for Microbial Research & and Science for Life Laboratory, Umeå University, 901 87 Umeå, Sweden

## Abstract

Physical virology investigates viral particles by focusing on their assembly, stability, mechanics, and interactions with host cells, neutralizing antibodies, and surfaces. Within this field, computational virology is becoming an indispensable pillar, serving as a “computational microscope” that bridges the spatio-temporal scales of viral processes, from individual protein dynamics to capsid assembly and cellular entry. This perspective article offers a critical overview of the current state, challenges, and future directions of computational approaches in physical virology. Our vision is anchored in the research presented at the 2025 EMBO/FEBS Lecture Course on Physical Virology held in Sant Feliu de Guixols, Spain, and complemented by a targeted survey among attendees. We survey the principal methodological frameworks in use, from all-atom to multiscale molecular simulations, mesoscale simulations, and growing integration of artificial intelligence (AI) tools. We also critically examine the central obstacles impeding the field’s progress, including the computational-experimental gap, limited accessibility to simulation data, reproducibility concerns, and systemic gender and geographic inequities. Finally, we outline future perspectives, proposing that integrating physics-aware AI with multiscale simulation frameworks, combined with community-driven data-sharing initiatives, will transform the computational microscope from a descriptive tool into a predictive engine for antiviral therapies, rational vaccine design, and biotechnological innovation.

## Teaser

This review highlights current methods in computational physical virology, ranging from all-atom and multiscale molecular simulations to mesoscale simulations, together with the growing integration of artificial intelligence (AI) tools. It also discusses the main challenges and future directions of the field across multiple spatial and temporal scales. Image created with the assistance of Gemini Pro and ChatGPT.

## Introduction

Physical virology investigates viruses as complex biomolecular machines, applying the quantitative principles of physics and chemistry to elucidate their biological operation. Its scope extends from the intrinsic physical and chemical mechanisms that govern viral particle self-assembly, stability, and mechanics to the critical intermolecular interactions that define host–pathogen recognition, cellular entry, immune evasion, and antibody neutralization. Moreover, infectious diseases pose a continuous threat, and addressing this multifaceted challenge requires a transdisciplinary perspective, uniting experts from complementary research areas. Physical virology embodies this approach, addressing processes across a vast range of spatio-temporal scales, from the folding, dynamics, and biomolecular interactions of single viral proteins (nanometers, nanoseconds) to the cooperative assembly of symmetric capsids (tens of nanometers, milliseconds to minutes) and the complex interplay between virions and host cells during entry and release (micrometers, minutes to hours). In recent years, computational methods have emerged as an indispensable foundation of this field. The “computational microscope” (Lee et al. 2009, Dror et al. 2012) can bridge these disparate scales. Physical experiments provide critical but often spatially or temporally limited snapshots of viral systems. These encompass high-resolution structural methods such as cryo-electron microscopy and nuclear magnetic resonance (NMR), dynamic imaging techniques such as single-particle tracking and super-resolution fluorescence microscopy, and biophysical assessments, including general spectroscopic tools, force spectroscopy, and biomolecular affinity measurements. In contrast, computational approaches serve as a generative engine for biological discovery, bridging these diverse and isolated data points into continuous mechanistic models. Computational approaches offer predictive models, simulate emergent behaviors, and help reveal the physical principles that shape biomolecular systems across space and time. For instance, they allow us to connect discrete experimental states, simulate the transient pathways between them, and reveal the underlying energetic landscapes that dictate biological function and evolution.

Viral processes are governed by a complex interplay of physical forces, including short-range hydrophobic effects during membrane insertion and entropic penalties during genome packaging. Among these, electrostatic interactions hold a uniquely fundamental role due to their long-range nature and profound sensitivity to physiological environments. Viruses are prime examples of polyelectrolyte systems, in which the interplay of charges dictates structure, function, and pathology (Zandi et al. 2020, Barroso da Silva et al. 2022). This recognition is not new; classic work by Perutz (1978), for instance, identified the critical role of electrostatic interactions in triggering the assembly of tobacco mosaic virus proteins. Since then, it has become evident that these forces are a recurring theme across virology. The interfaces of host–pathogen and antigen–antibody interactions are characteristically enriched with ionizable residues (Ramaraj et al. 2012, Qu et al. 2021), leading to the concept of “electrostatic epitopes” that extend beyond the immediate physical contact surface (Corrêa Giron et al. 2020, Poveda-Cuevas et al. 2020). Furthermore, the sensitivity of viruses to their environment is often mediated by electrostatics; pH, in particular, serves as a well-known biological trigger for key events such as host cell penetration and membrane fusion (Käsermann and Kempf 1996, Helenius 2013, Rawle et al. 2018), a mechanism exploited by viruses ranging from influenza (Lakadamyali et al. 2004) to flaviviruses (Poveda-Cuevas et al. 2022). It is worth noting that pH effects cannot be reduced to simple charge–charge interactions. Protonation events are intrinsically coupled to conformational changes through titration–dependent modulation. In addition, pH also regulates mesoscopic attractive forces that can drive complexation even on the “wrong” side of the isoelectric point (pI), due to charge fluctuations controlled by protonation equilibria, with membrane lipid composition providing an additional regulatory layer (Barroso da Silva et al. 2019, Yánez Arcos and Thirumuruganandham 2023, Guo et al. 2024, Poojari et al. 2025). The study of these electrostatic features is therefore not merely an academic exercise but is fundamental to understanding virulence and designing effective antiviral strategies and antibody therapies (Kiyoshi et al. 2014, Mason et al. 2021, Giron et al. 2023, Neamtu et al. 2023).

The historical trajectory of computational virology has been remarkable. It has evolved from early molecular dynamics (MD) simulations of isolated viral proteins, made possible by foundational software such as CHARMM (Brooks et al. 1983), to the current era of modeling entire icosahedral virus capsids and their packaged genomes at full atomistic detail. This progress is exemplified by landmark studies on the HIV-1 capsid (Perilla and Schulten 2017) and further illustrated by recent contributions exploring the conformational dynamics of the severe acute respiratory syndrome coronavirus 2 (SARS-CoV-2) spike variants (Milewska et al. 2025, Olivos-Ramirez et al. 2025, Cofas-Vargas et al. 2026) or the lipid architecture of the Zika virus (Tavares et al. 2026). Critically, this progress is characterized by a rich diversity of models, from highly detailed—but not necessarily equivalent—all-atom (AA) representations (Harrison et al. 2018, Singh et al. 2026) to more abstract coarse-grained (CG) approaches (Barroso da Silva et al. 2020, 2025, Machado and Pantano 2021, Lousa et al. 2022, Cofas-Vargas et al. 2024a, Poma et al. 2026).

The selection of a computational model is intrinsically linked to the scientific question. Since no single model can encompass all aspects of biomolecular complexity, progress depends on maintaining a broad spectrum of approaches (each optimized for different balances of computational efficiency, physical fidelity, accuracy, and user needs) (Barroso da Silva et al. 2020, 2025). This diversity empowers us to explore distinct biological questions with rigor and flexibility. While atomistic simulations provide unparalleled detail for studying specific molecular interactions, such as drug binding or the effects of a single mutation, CG models are essential for exploring larger-scale processes, such as the cooperative assembly of an entire capsid. This multi-resolution strategy is a hallmark of the field’s maturity. A synergistic triad of advances has propelled this progress: the exponential growth of high-performance computing (HPC) (Melo and Bernardi 2023), the development of more efficient algorithms and force fields at different levels (Melo and Bernardi 2023, Chipot 2024), and the concurrent explosion of high-quality structural data from experimental techniques (Kühlbrandt 2014). More recently, the advent of artificial intelligence (AI), particularly the revolutionary protein structure-prediction capabilities of AlphaFold (Abramson et al. 2024) and similar AI tools (Baek et al. 2021, Lin et al. 2023, Passaro et al. 2025), has profoundly reshaped the landscape of structural virology. These tools are accelerating hypothesis generation and enabling the structural and functional study of previously intractable protein complexes.

This review provides a critical perspective on the current status, prevailing obstacles, and future trajectory of computational methods in physical virology. Our analysis is specifically anchored in the themes, discussions, and cutting-edge research presented at the 2025 EMBO/FEBS Lecture Course on Physical Virology, “Physical Virology: across length scales”, held in Sant Feliu de Guixols, Spain, where leading researchers converged to define the key bottlenecks and future directions of the field. To ground this perspective in current community views, we conducted a targeted survey of experts. A prevailing theme that emerged was the view that computation’s primary role is to provide mechanistic explanations for processes that are experimentally elusive and to define what is physically plausible. Recognizing that “all models are bad, but some are useful,” this critical perspective is essential to the continued and rigorous advancement of the field. Notably, this work is not intended to provide a general or comprehensive literature review but rather to offer a timely overview grounded in feedback from the meeting.

As visually summarized in Fig. 1, following this introduction, the manuscript is organized to guide the reader from the current state of the field to its future horizons. In the next subsection, we begin with a snapshot of the contemporary landscape of physical virology, shaped by the collective insights from the lecture course. We then survey the state-of-the-art computational techniques, ranging from AA and super-coarse-grained (sCG) models to AI integration, which provide unprecedented mechanistic insights. We then illustrate the power of these methods through a series of case studies on diverse viral systems. Subsequently, we transition to a critical analysis of the primary challenges that impede progress, focusing on the computational-experimental interface, limitations in resources and training, and systemic imbalances within the community. We conclude by outlining key future perspectives, proposing actionable strategies to foster a more integrated, robust, and impactful future for the field.

**Figure 1:**
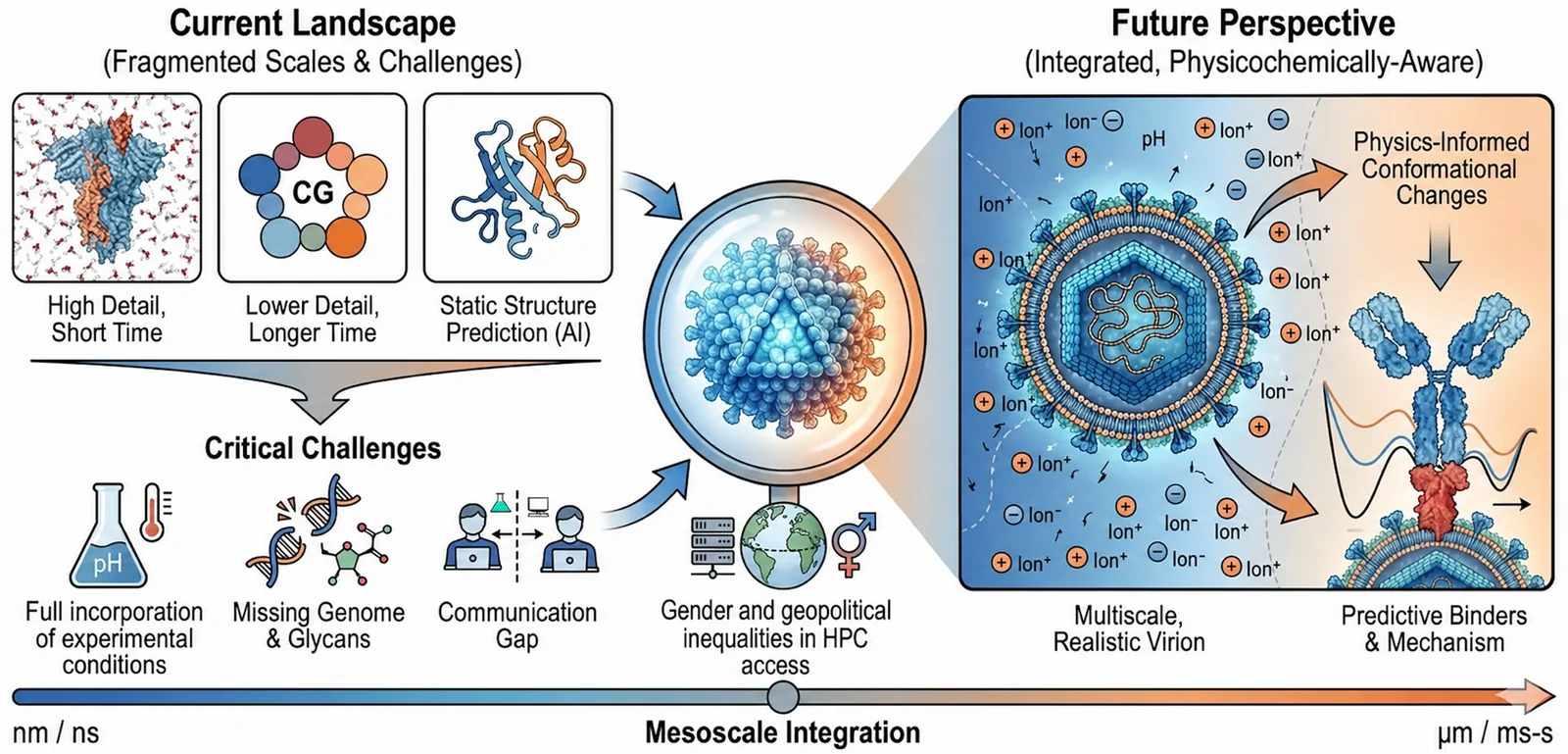
Graphical representation of the scope of the review (image created with the assistance of Gemini Pro and ChatGPT). The diagram summarizes the structural organization of this manuscript, outlining the logical flow from the contemporary landscape of physical virology to the exploration of state-of-the-art computational techniques across multiple scales, including AA, sCG, and AI models. Each of these approaches is presented in terms of the specific questions they are best suited to address, allowing researchers to cross lengths and timescales more effectively. The workflow further guides the reader through illustrative case studies, a critical analysis of current field challenges, including the computational and experimental interface, resource limitations, gender and geographical imbalance, and concludes with actionable future perspectives aimed at fostering integration within the community.

### A 2025 snapshot of the landscape of computational virology

We understand computational virology as the use of a battery of computational tools, including bioinformatics, modeling, simulation, and AI-based prediction techniques, to characterize the physical, structural, and functional features of viral systems. It is important to note that this conception goes far beyond merely performing simple models or simulations of viral molecules. For instance, most viral proteins are polyfunctional, coordinating complex, large quaternary structures in changing physicochemical environments. They are richer in polar amino acids with variable protonation states, and are coded by (hyper)variable genomes with overlapping reading frames, often read in both senses. Such complexity requires the deep integration of methods and expertise to provide consistent and significant insights currently inaccessible to experimental techniques. While computational virology has established itself as a broad and independent scientific discipline, it is intrinsically embedded within physical virology, bound by the shared goal of unraveling the molecular mechanisms that govern viral behavior and frequently relying on physical principles to do so. In practice, computational virology can be seen both as a core component of physical virology and, at times, as an independent branch that develops its own methodologies and perspectives adapted to the unique challenges of viral systems.

Computational virology was an important part of the 2025 EMBO/FEBS Lecture Course on Physical Virology, which showcased a field characterized by high thematic focus on physical aspects of virology from particle assembly to host-pathogen interaction, and maturing technical integration. Based on a thematic analysis of the 67 contributions presented (see Supplementary Information), SARS-CoV-2 and related coronaviruses remain a primary focus (∼25 works), underscoring the field’s central role in pandemic preparedness through studies of variant evolution and replication-transcription complexes, with particular emphasis on methylation enzymes such as nsp14 and nsp16. However, the scope is increasingly diversified. Research on alphaviruses (e.g. Chikungunya and Mayaro), flaviviruses such as Zika, and giant viruses such as Melbournevirus continues to push the boundaries of established biological length scales and structural complexity. Furthermore, the event highlighted significant progress in the study of icosahedral assembly and genome packaging in model systems such as the MS2 bacteriophage, the Brome Mosaic Virus, and the Nudaurelia capensis virus. The physical properties of therapeutic delivery vehicles also gained prominence, with several studies characterizing the nanomechanical stability and surface charge density of Adeno-associated virus capsids, African Swine Fever Virus antigens, and the Vault nanoparticle.

Technically, the computational approach is now deeply embedded in experimental workflows rather than serving as a parallel endeavor. Cryo-electron microscopy and tomography (cryo-EM/ET) remain the dominant structural benchmarks for resolving super-complex viral architectures. These are increasingly complemented by specialized biophysical tools, such as mass spectrometry (MS) for mapping assembly stoichiometries and interaction networks, and Atomic Force Microscopy (AFM) for assessing nanomechanical metastability. Critically, ∼22 works at the event explicitly combined theoretical or computational models with experimental data (such as integrating MD simulations with AFM force–distance curves), signaling a definitive shift toward integrative physical virology.

Approximately 42% of the works were presented by early-career researchers. Geographically, institutional metadata reveals a highly connected but uneven landscape concentrated in Spain, the USA, and Northern Europe. Spain (hosting the meeting) accounted for 22% of abstracts, Germany 15%, and Sweden 12%, forming a robust European core, while significant satellite clusters were observed in the USA and a minor one for Latin America. Moreover, 64% of the research originated from multidisciplinary centers, rather than traditional virology departments, underscoring physics and engineering as foundational rather than ancillary. While participation from researchers in Mexico, Brazil, Uruguay, and India is present, there is a visible tendency for groups in these regions to prioritize analytical theory or CG models. This trend corroborates our survey data (see later), in which 38% of respondents identified restricted access to HPC as a critical barrier, suggesting that infrastructure-driven constraints may inadvertently dictate the theoretical trajectories and model selection of researchers in the Global South (Santos and Pantano 2025). It is also worth mentioning that the “brain drain” from the Global South to the Global North is a massive reality in science. However, oftentimes, affiliation data masks global mobility and the migration of talent, making it difficult to obtain quantitative data about geographic distributions. This would be a critical dimension for a full analysis, which goes beyond the data currently available to us. Perhaps including records about country of origin in forthcoming conference application forms could help to fill this gap.

Furthermore, systemic imbalances are reflected in visibility metrics. While female researchers (including Nuria Verdaguer, Neha Kamat, Susan Daniel, Marta Bally, and Jodi Hadden-Perilla) occupied prominent roles as invited speakers (reaching ∼45%–50% representation at the plenary level), the community survey reflects a lingering perception that the specialized computational niche remains disproportionately male-dominated. A gender gap in senior authorship for computational-heavy projects remains evident (a ca. 3:1 male-to-female ratio), reflecting a “leaky pipeline” that the community is beginning to address. This diversity of targets and techniques is best exemplified by the following cutting-edge case studies on Bacteriophage HK97 (Coshic et al. 2024) and T7 (Aznar 2013), which represent the current frontiers of the “computational microscope” (see Section “The extremes of the computational microscope”).

## The state-of-the-art computational techniques

The field of structural virology has relied on static models of key protein domains and subsequent MD simulations at time and length scales characteristic of small systems (a few nm and microseconds) (Dror et al. 2012, Perilla et al. 2016). More recently, the computational field has evolved to incorporate multiscale methods that are enabling bridging the gap between molecular and biological timescales (Pak and Voth 2018, Coshic et al. 2024, Pérez-Segura et al. 2025). Such developments need to be integrated with experiments to understand the viral replication cycle. Although experimental methodologies such as cryo-EM and X-ray/NMR crystallography have become standard for elucidating isolated protein structures, they cannot capture the more dynamic, transient functional states (metastable) or conformations that characterize biological activity (Jiang and Tang 2017). In this context, computational methods, nowadays empowered by HPC, such as AA-MD, CG-MD simulations, time-independent thermodynamic approaches [e.g. Monte Carlo (MC) sampling and Poisson–Boltzmann (PB) continuum electrostatics], and more recently AI approaches, have become a cornerstone of physical virology (see Fig. 1). They serve as a true “computational microscope”, bridging static structural data with dynamic biological mechanisms, thermodynamic equilibrium states, and biomolecular interactions, thereby revealing how viral proteins fold, fluctuate, respond to electrostatic environments, and engage with partners across multiple scales (Pérez-Segura et al. 2025).

The paramount contribution of computational methods in physical virology is the delivery of mechanistic insight that cannot be experimentally isolated (Tarasova and Nerukh 2018). At the same time, simulations provide a unique sandbox for discovery, where physical interactions can be turned on or off, environments reshaped, and alternative scenarios tested, revealing how viral proteins and complexes behave under conditions that experiments cannot easily replicate. In navigating these approaches, it is crucial to distinguish the physical representation of the viral system (e.g. AA or CG models) from the simulation technique employed to explore it. A given model can be investigated either through time-dependent methods, such as MD, to track kinetic pathways, or through configuration-based stochastic methods, such as MC, to sample thermodynamic states. Building on this framework, the next sections present the current state of this diverse array of computational approaches, spanning from large-scale atomistic simulations and the transition to biological timescales via CG and mesoscale models, to the exploration of thermodynamic environments through mesoscale electrostatics and constant-pH simulations, and finally, the transformative role of machine learning (ML) approaches in physical virology.

To quantitatively assess how these diverse techniques are currently being deployed in the field, we analyzed the distribution of computational approaches presented at the 2025 EMBO/FEBS Lecture Course on Physical Virology (Table 1). Traditional time-dependent simulations remain a foundational pillar, with MD (ranging from AA to highly CG models) representing a significant portion of the studies. Complementarily, time-independent and continuum approaches, such as MC and PB solvers, carve out a specialized niche for exploring thermodynamic equilibrium, constant-pH ensembles, and mesoscale electrostatics. Notably, mesoscale approaches (grouped as “Other Methods” in Table 1) constitute the largest single category in the present analysis.

**Table 1:** Distribution of computational modeling and simulation approaches presented at the 2025 EMBO/FEBS Lecture Course on Physical Virology.

Computational methodological category	Participants/presenters	Frequency (*N*)
**Molecular dynamics (MD)** *(AA and CG)*	Coshic, Hadden-Perilla, Pantano, Aznar, Novev, Poma	6 (24%)
**Monte Carlo (MC)** *(Equilibrium and Kinetic)*	Barroso da Silva, Tyukodi, Strobl	3 (12%)
**Artificial intelligence (AI)** *(AlphaFold and Hybrid Models)*	Snijder, Khan, Prokopyeva, Sornaly, Schröder, Castorena, Borodavka	7 (28%)
**Continuum and other methods**(Poisson–Boltzmann*, Elastic Models, etc)*	Buzón, Menacho, Guzmán, Winkler, Botta, Díez Martínez, Biela, Golani, Bosch Fernández	9 (36%)
**Total**		**25**

Data was compiled from the official abstract book of the event (see Supplementary Information).

However, the most striking paradigm shift observed in this dataset is the amount of AI contributions. Surpassing traditional MD in frequency, AI approaches (predominantly driven by AlphaFold and related deep-learning structural prediction tools) now represent a dominant force. While this highlights the community’s swift adoption of ML for rapid structural modeling, it also reinforces the critical need to couple AI predictions with physics-based simulations (MD/MC) to validate whether these static, AI-generated snapshots represent true, possible biologically active conformations rather than the mostly static structural features that result from the reduction of ensemble populations to single, often symmetrized experimental models.

Building on this framework and these emerging empirical trends, the next sections present the current state of this diverse array of computational approaches. They span from large-scale atomistic simulations and the transition to biological timescales via CG and mesoscale models, to the exploration of thermodynamic environments through mesoscale electrostatics and constant-pH simulations, and finally, the transformative role of ML approaches in physical virology.

### From millions to billions of atoms: atomistic simulations in physical virology

The field of physical virology studies viral systems across time and length scales, linking molecular interactions to mesoscale structural and functional phenomena. Virions are ideal model systems because they bridge atomic-level dynamics with collective behaviors relevant to assembly and infectivity. Moreover, virion simulations often serve as methodological milestones in molecular modeling, driving advances in computational approaches. Figure 2 provides an overview of milestone simulations at different levels of detail. All-atom molecular dynamics (AA-MD) simulations represent the highest chemical resolution currently achievable in the classical domain of physical virology, capturing properties that are invisible to experimental probes (Pérez-Segura et al. 2025). The 2006 million-atom simulation of the satellite tobacco mosaic virus (STMV) established a landmark in computational virology, reporting the first simulation of a complete biological entity. Despite the ultra-short simulation times, they revealed the role of the RNA genome in the dynamical stabilization of the virion (Freddolino et al. 2006).

**Figure 2:**
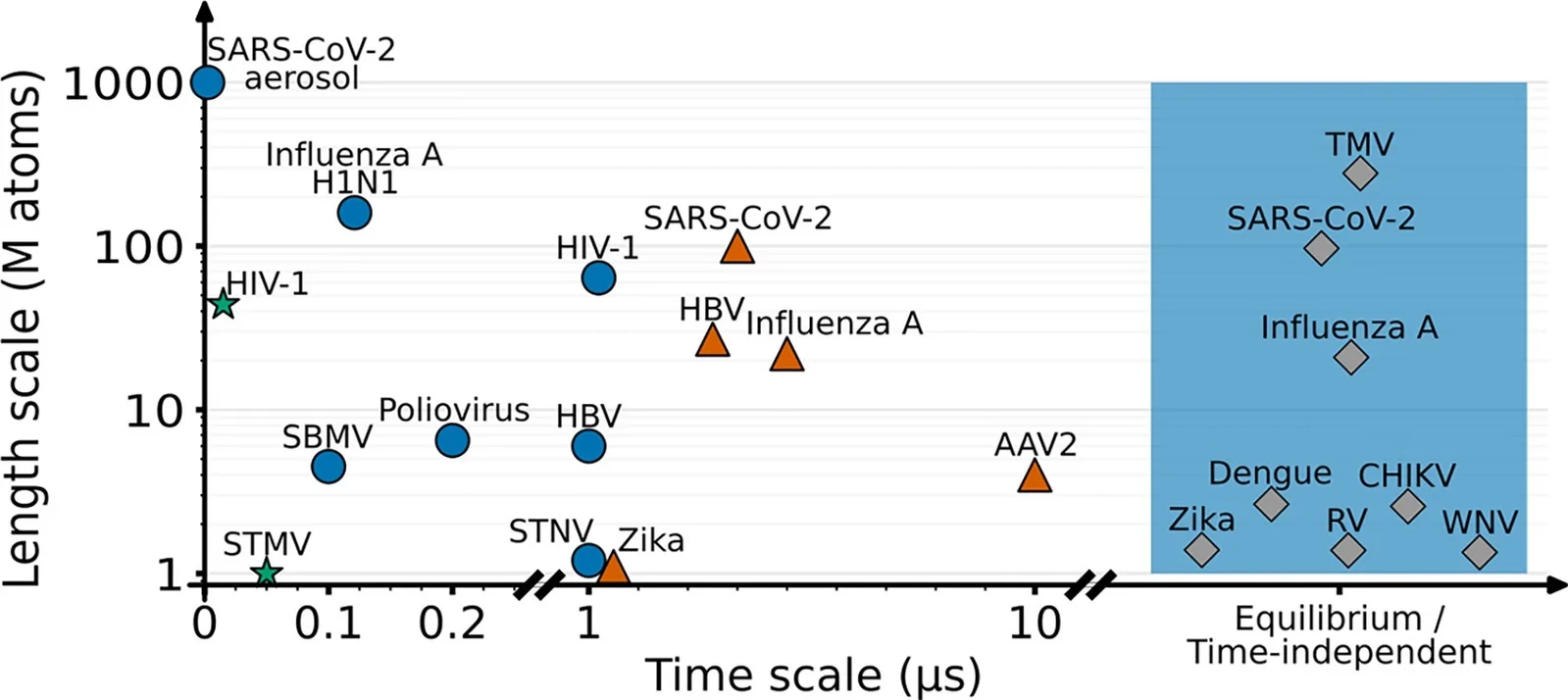
Computational studies in computational virology spanning different time and length scales in MD simulation are shown. Virion systems investigated using particle-based approaches are denoted by circles for AA-MD, triangles for CG-MD, and stars for ML-MD entries. Rhombs represent studies focusing on individual viral proteins (e.g. the SARS-CoV-2 spike and NS1 proteins from different flaviviruses) investigated using other computational approaches, such as PB calculations, constant-pH simulations, and AI-based tools.

Recent advances have pushed AA-MD simulations into the exascale regime, moving from isolated components to complete virions (Table 2). The 64M-atom simulation of the HIV-1 capsid revealed an asymmetric architecture with unique acoustic vibrational modes and hexagonal pores that act as selective gates for nucleotide translocation (Perilla and Schulten 2017, Jones et al. 2021). Another landmark system was the 160M-atom simulation of the Influenza A virion, showing how surface protein mobility dictates lipid distribution (Reddy et al. 2015), and the 1B-atom simulation of the SARS-CoV-2 aerosol model, which elucidated spike stability in respiratory droplets (Dommer et al. 2023).

**Table 2:** Milestones in large-scale AA-MD simulations for computational virology.

Virus system	*N* (Atoms)	Timescale (μs)	Key mechanistic insights	Reference
HIV-1 capsid	64 M	1.2	Asymmetric global dynamics and acoustic vibrational modes	(*Perilla and Schulten 2017*)
Influenza A virion	160 M	0.05	Lipid-protein coupling and bivalency of glycoproteins	(*Reddy et al. 2015*)
SARS-CoV-2 aerosol	>1 B	0.002	Environmental survival and spike stability in respiratory droplets	(*Dommer et al. 2023*)
HBV capsid	∼6 M	1	Discovery of rare, highly open conformational states	(Pérez-Segura et al. 2021)
Influenza A H1N1	∼160 M	0.121	Bind and transfer mechanism	(*Durrant et al. 2020*)
Satellite tobacco mosaic virus	∼1 M	0.050	RNA core stabilizes the capsid	(*Huber et al. 2017*)
Southern bean mosaic virus	∼4.5 M	0.100	Pentamers are the stiffest; stiffness depends on probe velocity.	(Zink and Grubmüller 2009)
Satellite tobacco necrosis virus	∼1.2 M	∼1	Calcium removal triggers STNV capsid swelling and increases water permeability 10-fold	(Larsson et al. 2012)
Poliovirus capsid	∼6.5 M	∼0.200	Poliovirus empty capsids function as semipermeable membranes	(*Andoh et al. 2014*)

Large-scale AA-MD simulation can identify rare functional states involved in infection. In the hepatitis B virus (HBV) capsid, microsecond-scale simulations uncovered an open conformation and a novel salt bridge (Pérez-Segura et al. 2021), dynamics information absent in static structures but essential for understanding how inhibitors impair viral assembly. By utilizing mesoscale simulations and Markov State Model theory, researchers uncovered the “bind and transfer” mechanism in neuraminidase. While the broader study spanned longer scales, the specific AA-MD trajectory of 121 ns provided the high-resolution detail needed to capture the discrete molecular transitions driving substrate movement (Durrant et al. 2020). AA-MD simulations of poliovirus demonstrated that the capsid exhibits selective permeability to water while remaining impermeable to ions. Additionally, interactions between internal ions along the interior wall generate a negative internal pressure that stabilizes the shell in the absence of a genome (Andoh et al. 2014, Pérez-Segura et al. 2025).

Simulations of satellite tobacco necrosis virus (STNV) revealed that removing structural calcium ions, mimicking cellular entry, triggers radial expansion and a 10-fold increase in water permeability. This process specifically initiates viral dissolution at the three-fold symmetry axis, highlighting how environmental changes drive infection (Larsson et al. 2012).

Despite advances in applying exascale computing for large-scale applications in physical virology, a severe timescale limitation remains: many viral systems undergo large conformational changes on timescales beyond the microsecond range.

### Reaching biological timescales: CG simulations

Notwithstanding the capabilities of AA-MD, several viral processes occurring on the millisecond-to-second timescales, such as self-assembly and extensive maturation transitions, remain computationally unfeasible for atomistic models (Huber et al. 2017). To overcome this limitation, CG-MD simulations (Huber et al. 2017, Machado et al. 2017, Poma et al. 2021, Souza et al. 2025, Mandal et al. 2026) can reduce the system complexity and reach longer timescales (Table 3). CG-MD simulations have demonstrated that viral capsid assembly is a dynamic, reversible process; the capacity of subunits to dissociate is essential both for viral uncoating during entry, and for achieving the correct assembly process and avoiding kinetic traps (Rapaport 2004). CG models serve as an essential connection by generalizing empirical observations across various experimental systems (Hagan and Zandi 2016).

**Table 3:** Milestones in large-scale CG-MD simulations for physical virology.

Virus system	*N* (beads/atoms)	Timescale (μs)	Key mechanistic insights	Reference
SARS-CoV-2 envelope	20-30 M/∼100 M	4	M dimers form filament-like macromolecular assemblies	(*Pezeshkian et al. 2023*)
SARS-CoV and SARS-CoV-2 envelopes	7.3 M/∼29 M	3–12	S proteins exhibit high dynamics; specific lipid-binding preferences at TMD sites stabilize the architecture.	(Wang et al. 2022)
HBV virion	∼3 M	3.5	Capsid spikes stabilize S-protein transmembrane domains; lipid diffusion within the envelope slowed 5x	(*Urano and Shinoda 2026*)
AAV2 capsid	∼0.9 M/∼3.7 M	10	Binding induces GM3 lipid clustering and membrane curvature; AAVR flexibility is revised in the membrane context.	(Pipatpadungsin et al. 2024)
Zika virus	1.5 M/8 M	2	Anionic phospholipids play a role in stabilizing the envelope	(*Soñora et al. 2021*)
Influenza A virion	∼5.5 M/22 M	5	Restricted lipid mobility increases environmental stability.	(*Reddy et al. 2015*)

In addition to the self-assembly process in viral capsids, CG models have elucidated intricate interactions between virus and host membranes. Simulations employing the Martini 3 force field (Souza et al. 2021) have explored the interaction between adeno-associated virus serotype 2 (AAV2) and its universal receptor; these ∼0.9 million bead systems (∼3.7 million atoms), simulated over 10 μs, demonstrate that capsid binding prompts local membrane curvature and the recruitment of specific lipids such as monosialodihexosylganglioside (Pipatpadungsin et al. 2024). Within this environment, AAVR flexibility is revised in the membrane context. Similarly, the SIRAH force field (Klein et al. 2023) offers a multiscale framework that enables virion-scale simulations, such as those for Zika, which have revealed that phospholipids play a critical role in stabilizing the viral envelope (Soñora et al. 2021) and attributed an active role of the membrane coat to store tensile energy (Soñora et al. 2022). Other aspects explored by CG-MD include the nanomechanical stability of SARS-CoV-2 spike protein variants and their interactions with engineered nanobodies and antibodies (Cofas-Vargas et al. 2024b, 2026, Milewska et al. 2025).

Supramolecular organization is further clarified in massive virions such as the SARS-CoV-2 envelope (20–30 million beads/∼100 million atoms), where M-dimer transmembrane domains (TMDs) preferentially form filament-like assemblies over 4 μs (Pezeshkian et al. 2023). Comparative studies of SARS-CoV and SARS-CoV-2 envelopes (7.3 million beads/∼29 million atoms) simulated over 3–12 μs show that S proteins exhibit high dynamics and specific lipid (PI/CHOL) binding preferences at TMD sites to stabilize the architecture (Wang et al. 2022). These studies highlight how viruses modulate their immediate biophysical environment: in both the HBV virion (∼3 million beads reaching 3.5 μs) and Influenza A virion (∼5.5 million beads/22 million atoms over 5 μs), protein-mediated tethering reduces lipid mobility five-fold compared to simple lipid vesicles (Reddy et al. 2015, Urano and Shinoda 2026). This restricted mobility likely confers physical robustness and environmental stability, demonstrating the utility of CG-MD in uncovering the active regulation of viral structural integrity.

Finally, a relatively less developed area involves the combined use of quantum, fully atomistic, and CG methods to simulate complex viral assemblies. In these cases, modeling and simulations can be performed separately, feeding one simulation framework with the results of higher- or lower-granularity representations (Viso et al. 2018, Coshic et al. 2024, Palur et al. 2026), or simultaneously, with different granularity regions talking to each other within the same simulation (Zavadlav et al. 2014, Machado et al. 2019, López et al. 2022). Although these techniques hold exciting promise, current limitations in available software are still hampering their widespread use in computational virology.

### Lower granularity and mesoscale models for viral assembly and mechanics

The complexity of viral systems, spanning multiple lengths and timescales, has driven the development of a diverse set of CG approaches. Beyond the atomistic and residue-level approaches described in the previous paragraphs, coarser or mesoscale modeling, simplified models are used to achieve a broader spatiotemporal coverage.

At the coarsest end of the modeling spectrum, low-granularity CG approaches represent entire capsid subunits or even multiple residues as single particles, drastically reducing the degrees of freedom of the system. Particle sizes ranging from one bead per capsid subunit to one bead per amino-acid residue have been employed in the study of viral capsids (Arkhipov et al. 2006, Hagan and Chandler 2006, Nguyen et al. 2007, Perlmutter et al. 2013, Wagner and Zandi 2015, Panahandeh et al. 2020). A prominent class of such models is the so-called “shape-based”, in which few beads represent each protein unit, with the number of beads matched to the protein shape and model parameters derived from crystal structures. This approach has been applied to study the stability and mechanical properties of STMV and HBV capsids under AFM indentation (Arkhipov et al. 2006, Arkhipov et al. 2009, Roos et al. 2010). At a similar level of description, rigid capsid subunit models have been used to investigate the general principles of capsid (mis)assembly, and the role of environmental conditions such as temperature and protein concentration (Nguyen et al. 2007, 2009). Another widely used strategy represents capsid proteins as a single particle, as employed by Qiao et al. (2015) in MC simulations of HIV capsid assembly intermediates, suggesting that trimers of dimers constitute a stable preassembly intermediate.

Elastic network models (ENMs) offer a further simplification, representing the capsid building blocks as an interconnected network of harmonic springs. Despite their simplicity, ENMs have been shown to reproduce thermal fluctuations, elastic properties, and assembly pathways in close resemblance to atomistic and more detailed CG simulations (May and Brooks 2011, Panahandeh et al. 2020). These models have been applied, for instance, to study HBV capsid assembly and its dimorphism, revealing how the ratio of quasi-equivalent dimer types in early assembly intermediates determines the final assembly pathway (Mohajerani et al. 2022).

Among the most widely used mesoscale frameworks are Brownian dynamics (Li et al. 2014, Islam et al. 2017), which captures the diffusive behavior of viral particles and their components by treating the solvent implicitly and incorporating stochastic forces. Similarly, dissipative particle dynamics (DPD) (Liu et al. 2012, Moreno et al. 2020, Chen 2022) represents both solute and solvent as soft interacting beads, enabling the simulation of hydrodynamic effects at scales inaccessible to MD. An extension of this framework, smoothed dissipative particle dynamics (SDPD) (Español and Revenga 2003), combines the thermal fluctuations of DPD with the spatial discretization of smoothed particle hydrodynamics, providing a thermodynamically consistent description of mesoscale fluid-structure interactions particularly suited to the study of biological particles suspended in viscous media. MC simulations (Li et al. 2019) and CG polymer models (Debets et al. 2020, Ilnytskyi 2020) have also been employed to investigate viral assembly and membrane interactions at a significantly reduced computational cost.

At an even coarser level of description, rigid bead-rod models (Kanso et al. 2020, Kanso et al. 2021, Chaurasia et al. 2023) and rigid multi-blob methods (Moreno et al. 2022) represent viral particles as assemblies of geometric objects, allowing the cost-effective computation of translational and rotational diffusion coefficients from hydrodynamic theory alone. These approaches have proven especially useful for exploring how virion morphology—including the size, shape, number, and spatial distribution of surface spike proteins—governs passive viral transport (Moreno et al. 2022).

While high–resolution simulations dominate the literature, the 2025 EMBO/FEBS Lecture Course on Physical Virology highlighted the indispensable role of highly efficient, yet less frequently employed, models. A standout perspective was provided by Maria Aznar Palenzuela, whose “super–coarse–grained” (Aznar 2013) approach pushes the limits of abstraction by representing entire pentamers as single beads. When discussing the necessity of such simplification to reach truly biological assembly timescales, Aznar captured the essence of the field’s pragmatism during her presentation, responding to questions with the remark: “I am an economic person!” This “economy of scale” is not merely a computational convenience but a theoretical requirement to effectively bridge the gap between single–molecule kinetics and the stochastic assembly of the whole virion.

Collectively, these low-resolution methods occupy a critical niche in the computational virology armamentarium, enabling the investigation of phenomena that emerge at the scale of the whole virion. These include diffusion, capsid assembly, surface protein dynamics, and clustering, which remain beyond the practical reach of fully atomistic simulations.

### Thermodynamic landscapes: mesoscale electrostatics and constant-pH simulations

A major challenge in simulating viral mechanisms is that standard coarse-grained molecular dynamics (CG-MD) approaches, although excellent at capturing structural choreography, classically treat protonation states as static. This can be a significant limitation for understanding viral life cycles, which are intrinsically driven by pH gradients and dynamic charge fluctuations. To overcome this, mesoscale rigid–body simulations based on the MC method in the semi-Grand Canonical ensemble [e.g. FORTE (Barroso da Silva 2024) and PROCEEDpKa (Poveda-Cuevas et al. 2020) frameworks] have emerged as critical tools for mapping the “electrostatic landscape” of viral proteins. The PROCEEDpKa method (PRediction Of eleCtrostatic Epitopes basED on pKa shifts) is particularly valuable for identifying so–called “electrostatic epitopes”, a broader view of epitopes in which inner titratable groups also participate in the interplay of interactions with antibodies, as demonstrated for the NS1 protein of West Nile virus (WNV) and its Fab fragment 22NS1 under different conditions (Poveda-Cuevas et al. 2020). Complementarily, the FORTE approach (Fast cOarse–grained pRotein–proTein modEl) enables the estimation of relative binding affinities as a function of pH, providing a powerful tool to explore how protonation dynamics modulate macromolecular recognition and complex formation (Barroso da Silva 2024).

A key advantage of these approaches is their ability to incorporate the charge-regulation mechanism, i.e. the dynamic adjustment of a protein’s net charge in response to environmental changes or to the proximity of other macromolecules. This phenomenon is particularly relevant in the so–called “wrong–side–of–pI” regime, where macromolecules bearing charges of the same sign can nevertheless attract and form complexes (Lunkad et al. 2022, Barroso da Silva 2024). In such cases, charge regulation becomes essential for explaining protein–macromolecule interactions that defy classical electrostatic expectations. Moreover, accounting for pH effects is crucial, as protonation dynamics often act as molecular switches that modulate viral assembly, stability, and host interactions.

Recent studies on SARS–CoV–2 Spike protein variants (Alpha through Omicron) revealed a direct linear correlation between the net charge of the receptor binding domain (RBD) and its binding affinity for the angiotensin-converting enzyme 2 (ACE2) (Barroso da Silva et al. 2022). This so–called “RBD charge rule” serves as a predictive compass for viral transmissibility, showing that new variants tend to evolve toward increased positive electrostatic potential to favor interaction with the negatively charged host receptor. Tests with the most recent variants have confirmed this behavior, reinforcing the robustness of the rule (Barroso da Silva et al. 2025).

Constant–pH (CpH) simulations have further elucidated pH–dependent “molecular switches” in flaviviruses. For the NS1 protein of Zika and Dengue, CpH MC simulations demonstrated that self–association into dimers and tetramers is strictly regulated by pH–sensitive van der Waals and Coulombic balances, providing a molecular basis for diagnostic cross–reactivity and identifying novel antigenic hotspots (Poveda-Cuevas et al. 2022, Valotto et al. 2026). In antibody engineering, these features have been applied to optimize monoclonal antibodies, such as CR3022, by suggesting mutations that enhance electrostatic complementarity, even against highly mutated variants such as Omicron (Corrêa Giron et al. 2020, Neamtu et al. 2023).

In contrast to exascale MD, the continuum-based work of Guzman and collaborators utilizes a generalized electrostatic framework based on the Poisson-Boltzmann (PB) equation to characterize viral interactions at the mesoscale. Through a “tomography-type” analysis of the Zika capsid’s icosahedral symmetries, this approach quantified subnanometric interactions with flat substrates, pinpointing specific amino acid clusters that drive adsorption (Cooper et al. 2022). Furthermore, the model explored RNA polarization under confinement, revealing how the genome’s spatial organization responds to external pH and salinity gradients. Expanding on this framework to enveloped viruses, recent work by Guzman and Menacho utilized the PB equation to define the complex virus-material interface of SARS-CoV-2 variants, mapping how their surface proteins deform and adsorb onto different substrates (Bosch et al. 2024, Bosch-Fernández et al. 2026). Collectively, these insights provide a quantitative basis for designing virucidal surfaces and biosensors, in which charge density can be tuned to capture or neutralize specific viral symmetries and surface topologies.

### Machine learning approaches in computational virology

Recent advancements in foundational ML models based on neural networks have enabled the simulation of viral structures with quantum-level precision at the exascale. The “Allegro force field” has been successfully implemented to simulate a fully explicitly solvated HIV-1 capsid comprising 44 million atoms. This landmark simulation, conducted on the Perlmutter supercomputer, exhibited stable dynamics over nanosecond timescales, successfully reconciling the trade-off between precision of first-principles quantum mechanics and the scalability necessary for extensive biological assemblies (Kozinsky et al. 2023). Complementing this HPC methodology, GRAPPA (Graph-Attentional Neural Network) was used to produce a stable simulation of an entire STMV virus comprising ~1 million atoms on a single GPU (Seute et al. 2025). ML force fields move beyond simple observation by bridging the gap between quantum-level accuracy and biological scalability. Unlike classical models that rely on rigid “ball-and-spring” approximations, ML potentials are trained on ab initio data to predict complex energy landscapes with near-quantum precision (Kozinsky et al. 2023; Seute et al. 2025).

This approximation allows researchers to capture chemically reactive events, such as bond breaking and electronic polarization, which are invisible to traditional simulations but essential for understanding viral stability and dynamics (Cui et al. 2025; Poma et al. 2026). Although highly promising, this emerging ML–based approach was not presented at the 2025 EMBO/FEBS Lecture Course on Physical Virology, likely reflecting its very recent development and the fact that it is not yet widely incorporated into mainstream computational virology workflows.

The recent emergence of AI–driven structural tools, such as AlphaFold and Boltz–2, has been heralded as revolutionary, providing high–quality starting models for previously intractable viral complexes (Pérez-Segura et al. 2025). Nevertheless, a critical gap remains in the community’s understanding of its inherent limitations (Santos and Pantano 2025). While these AI tools are proficient at delivering a static “parts list” of viral proteins, they fundamentally lack the capacity to account for conformational heterogeneity and the rare functional states that govern viral regulation (Pérez-Segura et al. 2025). Consistent with feedback from participants in the 2025 EMBO/FEBS Lecture Course on Physical Virology (see below), viewing AI as a replacement for experimental validation is a precarious misconception. These models lose predictive power in the absence of high sequence similarity or when navigating the “dark proteome” of emerging pathogens. This is particularly the case for RNA viruses, which often lack the evolutionary features that lie at the basis of AI algorithms or have very scarce structural information available. A more profound limitation lies in the inability of current AI architectures to incorporate environmental physicochemical parameters. Since training datasets are derived from the Protein Data Bank (PDB) (Berman et al. 2000), they inherit an intrinsic “crystallization bias”. AI models interpret structures solved under disparate non–physiological conditions as universal benchmarks. This can be a dangerous oversimplification that risks misrepresenting biologically relevant states (Saldaño et al. 2022). For example, one may obtain a pre–fusion state when the biologically relevant conformation is post–fusion, a transition tightly regulated by pH–dependent electrostatic triggers (Harrison 2008, Rey and Lok 2018). This might be particularly deleterious when modeling viral glycoproteins and antibody–antigen (Ab–Ag) interactions, where subtle conformational shifts dictate neutralization efficacy. In this particular arena, despite progress in Protein Language Models, the success rate for predicting Ab–Ag interfaces remains modest (Clifford et al. 2022, Cia et al. 2023). This low performance stems not only from the paucity of structural data for these complexes but also from the systemic neglect of the physicochemical context during training. Our previous analysis of the current structural landscape reveals that the average pH for solved complexes is 7±1, with some structures determined at biological extremes (pH 3.3–11) (Grandguillaume et al. 2025). Current AI models are blind to how these conditions modulate the net charge of titratable residues and, consequently, binding affinity. To bridge this gap, simulation methods such as those discussed above are indispensable, enabling the quantitative incorporation of physicochemically relevant information and the correction of AI–generated models within biologically relevant pH regimes.

Conversely, as the field moves toward the exascale era, the integration of ML and multiscale modeling will be pivotal in elucidating the comprehensive dynamic mechanism of viral infection (Poma et al. 2026). The primary objective is to attain physiological authenticity, in which simulations of complete virions with heterogeneous envelopes and encapsulated genomes yield the mechanistic insights necessary for the development of next-generation antivirals such as Lenacapavir (Pérez-Segura et al. 2025). Future success depends on bridging the communication gap between experimentalists and theorists to ensure that simulations begin from biologically relevant initial structures.

### The extremes of the computational microscope

To illustrate the methodological diversity detailed in the preceding sections, we highlight two case studies presented at the meeting. These examples represent the opposing frontiers of computational resolution, from exascale AA simulations to extreme coarse-graining, demonstrating how different physical scales require tailored computational philosophies:

1. Exascale AA simulation (Bacteriophage HK97): The work by Coshic and colleagues exemplifies the current limits of AA-MD simulation (Coshic et al. 2024). Employing a multi-resolution approach to mimic a viral packaging motor, the team reconstructed the complete virion, generating a final 26-million-atom model that encompasses the 39 732-base-pair genome, internal water, and ions. Microsecond-scale simulations revealed that the DNA is packaged via a loop extrusion mechanism. This finding represents a mechanistic milestone, as it challenges the historical dogma that DNA in these phages strictly adopts a coaxial spool arrangement, while also elucidating elusive physical properties.
2. Super-coarse-grained models (Bacteriophage T7): At the opposite end of the resolution spectrum, the research presented by Aznar demonstrates the power of massive simplification (Aznar 2013). To bridge the gap between virtual simulations and experimental AFM of the Bacteriophage T7, her group employs a “super-coarse-grained” approach where entire pentamers are represented as single beads. This extreme abstraction is not a limitation, but a necessity to reach the macroscopic timescales required to observe mechanical deformation and large-scale assembly. Embodying the pragmatic philosophy that computational virology must be “economic” to be biologically relevant at the mesoscale, this approach provides the direct theoretical validation needed to interpret experimental nanomechanical data.

## The survey

To ground this perspective in current community views, we conducted a targeted survey among experts attending the event. Participation was entirely voluntary, with all participants invited via email to complete a structured questionnaire. The objective was to quantify the community’s perceptions regarding the primary roles, critical bottlenecks, and future directions of computational virology. The previous paragraphs offer an overview of landmark studies in computational virology, providing context for this survey. Our discussion draws on survey data representing participants across career stages, from PhD students to senior group leaders.

The questionnaire consisted of four core questions designed to capture both quantitative and qualitative insights:

1. (**Q1**) Based on your experience, how would you describe the current role of computational approaches in advancing the field of physical virology?
2. (**Q2**) To what extent do you already apply or foresee computational methods (bioinformatic tools, molecular simulations, and AI) contributing to your own research goals in virology? If applicable, please describe the specific objectives or expected outcomes of integrating such approaches.
3. (**Q3**) What do you perceive as the main challenges or limitations to the effective integration of computational approaches with experimental work in virology today?
4. (**Q4**) Independent of your answer to Q3, do you think the integration of computational and experimental methods is hampered by deficiencies in gender, geographic, or other imbalances in science?

Responses were anonymized. Names and email addresses were collected solely for internal tracking and were not disclosed in any analysis. This design ensured that the survey captured candid perspectives while safeguarding participant confidentiality. Importantly, we did not take part in the survey ourselves, to avoid biasing the results and to keep them as independent as possible from our personal impressions. Nevertheless, we noted that the responses corroborated our observations from informal conversations with participants during the event, reinforcing the survey’s validity as a reflection of the community’s collective views.

An important point to note is that this survey received 24 responses from a total of 108 invited participants (23% response rate). The results presented here represent the opinions of this specific group of respondents and may not fully reflect the views of all invitees, due to the potential risk of non–response bias.

The survey results (see Fig. 3) suggest the community highly values the role of computational methods. In quantitative terms, the main role of computer simulations in physical virology, as perceived by the colleagues, is to provide mechanistic insight. This option accounts for up to 75% of responses and involves revealing hidden physical aspects of viral lifecycles, such as internal pressure stabilizing empty capsids. Second, 17% of the community recognizes these methods as valuable tools for transforming static snapshots into dynamic functional models, helping identify fleeting interactions that are not obvious from static experimental structures. Finally, 8% of respondents consider simulations a predictive tool for estimating molecular behavior before wet-lab validation. Hence, the general opinion seems to suggest that the experts find computational tools to be a physics-based filter to distinguish energetically plausible pathways from artifacts during rapid transitions that imaging cannot resolve. This consensus underscores that, far from being auxiliary, computational approaches are increasingly regarded as indispensable in guiding experiments, reducing trial–and–error, and accelerating the discovery of mechanistic principles that would otherwise remain hidden.

**Figure 3:**
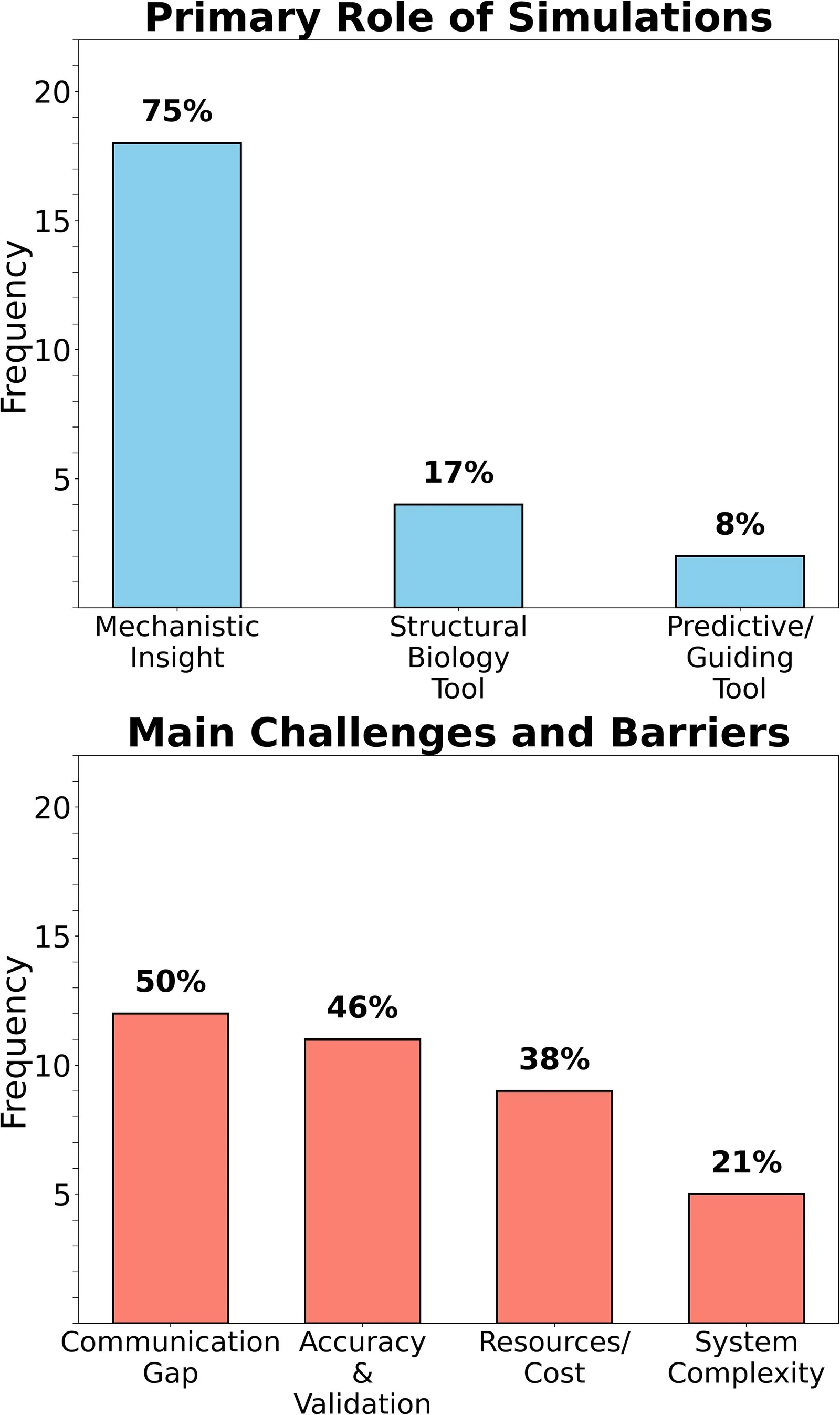
Main roles and challenges of biomolecular simulations in physical virology. Based on the 2025 EMBO/FEBS Lecture Course on Physical Virology expert survey (*N* = 24). The charts summarize the primary roles attributed to biomolecular simulations (top), highlighting a strong consensus that they provide mechanistic insight to explain experimental observations. The bottom panel shows the systemic barriers currently facing the physical virology community, emphasizing limitations in data interpretability and technical capabilities as the most significant bottlenecks for the acceptance of biomolecular simulations as a predictive biophysical tool.

When asked about possible barriers that limit the assimilation of experimental and computational methods, the survey identifies several hurdles. The most frequent answer (50%) regards the integration with biophysical experiments, which is often limited by communication gaps that hinder collaborative workflows. In second place, the difficulties in proper validation and accuracy (46%), where discrepancies in biophysical scales between simulations and experiments reduce confidence in computational predictions. Notably, in third place, we find the high cost and unequal access to exascale computing resources (38%). This is indeed a significant issue that limits the effective access to cutting-edge techniques able to effectively bridge the gap between the affordable sizes and timescales of simulated systems and experimental resources. Finally, the challenges imposed by the complexity of viral systems received 21% of the answers. In this regard, researchers recognize viruses as highly complex systems that extend beyond proteins and solvent to include additional cofactors, such as chemical modifications, glycosylation states, and RNA, which are typically not included in computer simulations. Some of these limitations are further discussed in the next paragraph.

## Critical challenges at the computational-experimental interface

Some of the challenges identified by the community regard limitations associated with the accuracy of the methods or the impossibility of achieving proper experimental validation of computational predictions. Weaknesses associated with limited accuracy are multifactorial. The computational cost of the calculations often compromises the statistical significance of the simulations, as they rely on single runs of relatively large molecular systems. These studies are conducted under the strong assumption that simulations actually sample a, if not ergodic, sufficiently broad conformational space. However, because the viral proteins are multifunctional and can have different functional roles at varying pH, local concentrations, glycosylation states, and in the presence of a variety of interactors, the ergodicity hypothesis can be severely compromised. Moreover, a significant point of concern when simulating whole virus particles is the lack of information about the genome conformation inside the capsids or envelopes. Because genomes are either absent or their coordinates are blurred by symmetry operations during the refinement, initial systems are usually constructed using only the 3D structures of capsids or envelopes filling the inner space with solvent. Therefore, simulations might be more comparable to virus-like particles than to virions.

Because computational virology is a relatively new area with few groups worldwide, there is still a need to establish unified best practices, standardised pipelines, readily accessible simulation protocols, and specific analysis tools. This is particularly important, given the significant cost of producing and storing the large volumes of data generated by the simulations (Riccardi et al. 2019). In this aspect, increased adherence to the FAIR (Findable, Accessible, Interoperable, Reusable) principles in biomolecular simulations promises to greatly advance the field (Amaro et al. 2025). Making the data available would not only contribute to exploiting it to its full potential, it would also allow groups in disadvantaged regions with no access to HPC to access cutting-edge research, increasing the diversity.

As evident from the previous paragraphs, there are a number of possible methodological compromises that require deep consideration and caution to evaluate the result of computational studies. So, getting the most out of computational studies in physical virology requires a deep understanding of the subtleties of computational techniques, together with deep insights into molecular and structural virology. This complex scenario requires specific training and courses. Solving this requires a continuous community effort, and we believe that scientific events such as the 2025 EMBO/FEBS Lecture Course on Physical Virology make relevant contributions to reducing communication gaps and increasing interdisciplinarity. Still, special attention should be paid to facilitate access to geographically disadvantaged regions not only by granting fellowships, but also by including speakers from those regions in the scientific programs and organizing events in the Global South. Below we address some of the main challenges the computational virology community faces nowadays.

### The glycosylation challenge

Viral glycoproteins typically carry large amounts of carbohydrate residues, sometimes accounting for as much as 50% of the molecular mass of envelope glycoproteins (Mascola and Montefiori 2003). It is increasingly evident that these post-translational modifications fulfill critical biological functions, not only shielding the virus particle from immune recognition but also tuning the viral replication cycle (Vigerust and Shepherd 2007). For example, it has been proposed that viral protein glycosylation (specifically the presence of O-glycan clusters) can modulate Herpesvirus interaction dynamics with negatively charged glycosaminoglycans in the host glycocalyx. Presumably, modulation occurs via a delicate adjustment of binding site exposure, combined with steric and electrostatic repulsion effects (Sun et al. 1996, Coleman et al. 2003, Altgärde et al. 2015, Delguste et al. 2019, Trybala et al. 2021). Specific glycosylated residues have also been proposed to modulate viral fusion, as shown for Henipaviruses (Olofsson et al. 2023, Stone et al. 2016), possibly through altered protein conformations. In both cases, this primarily experimental work would greatly benefit from computational validation of the underlying physical mechanisms. Similarly, in the case of SARS-CoV-2, the extended heparan sulfate binding pocket on the spike protein (Kim et al. 2023) has been computationally shown to be partly shielded by neighboring N-glycans, suggesting a modulatory role of spike glycosylation on host cell interactions, although experimental evidence is still lacking.

From these examples, it is evident that comprehensively modeling viral systems ultimately requires accounting for both the amino acids and their complex carbohydrate coats. However, the challenges are immense, given that protein glycosylation is highly heterogeneous and dependent on cellular conditions, while putative glycosylation sites may or may not be occupied. Consequently, the vast majority of studies, both computational and experimental, often deliberately omit these structures. Computationally, the highly flexible and dynamic nature of glycans requires extensive conformational sampling to achieve statistical convergence, which remains a profound bottleneck when simulating large biomolecular assemblies. In addition, current AA descriptions that include glycan contributions, such as GLYCAM (Kirschner et al. 2008) or CHARMM (Guvench et al. 2011) force fields, still face limitations in extensively sampling conformational changes in proteins and glycan flexibility, and alongside inherent empirical inaccuracies in glycan–protein interaction energetics, further limiting their use in large-scale simulations. Due to the size, heterogeneity, and intrinsic flexibility of glycans, AA-MD simulations remain computationally demanding. To address these challenges, CG approaches at different levels of resolution have been developed at the price of inevitably sacrificing fine-grained details, which can exacerbate underlying force field inaccuracies by, e.g. losing specific hydrogen-bonding directionality and other fine steric interactions that can be crucial for glycan recognition. In this context, CG modeling enables the simulation of larger systems over longer timescales. CG approaches are particularly well suited to studying the dynamics of highly glycosylated proteins and their interactions with proteins, membranes, and other biomolecules (e.g. glycosaminoglycans). Several models have been proposed that balance structural accuracy with computational efficiency (Samsonov et al. 2015, Danielsson et al. 2023, Garay et al. 2024). These approaches differ in their treatment of glycosidic linkages, branching, and electrostatic features, but collectively highlight the importance of multiscale strategies. In particular, force fields such as Martini 3 and SIRAH provide unified frameworks for multicomponent simulations of proteins and glycans (Grünewald et al. 2022, Lutsyk et al. 2022), enabling the study of glycan–protein interactions and associated conformational changes while improving transferability and scalability compared to purely atomistic descriptions. Experimentally, high-resolution X-ray crystallography frequently requires the enzymatic cleavage of flexible glycans to achieve stable crystals. Similarly, many biomolecular interaction studies using, e.g. surface plasmon resonance or bio-layer interferometry have often been carried out with partially glycosylated or non-glycosylated recombinant proteins. This highlights that many core biomolecular interactions can be studied independently of the glycan shield, even though glycans may play critical biological roles.

Furthermore, cellular glycans are of prime relevance in the viral life cycle. Many viral pathogens take advantage of the host glycocalyx (the dense, heterogeneous layer of carbohydrates coating the cell) to accumulate and initiate infection. Since the glycocalyx can also serve as a barrier against pathogen invasion through premature virus entrapment, the virus particle must exhibit a finely tuned dynamic interaction profile with these diverse structures to ensure efficient transfer to an entry receptor or transport toward the entry site. Such multiscale processes remain mechanistically poorly understood and would greatly benefit from computational modeling.

Nevertheless, the field (including many works presented at the 2025 EMBO/FEBS Lecture Course on Physical Virology) still largely follows a point-to-point approach, dissecting individual aspects in isolation. Rather than a mere technical limitation, this reductionist strategy is a pragmatic first step in physical virology. Temporarily omitting these highly dynamic and heterogeneous layers allows researchers to isolate variables and achieve a baseline understanding of the core thermodynamic mechanisms driving protein–protein interactions. Accordingly, point-to-point approaches remain indispensable for establishing baseline molecular mechanisms. Yet, only by progressively integrating viral glycosylation and host glycocalyx interactions can computational virology achieve a complete mechanistic picture of viral entry (Koehler et al. 2020).

### The communication gap and interdisciplinary training

The most pervasive challenge, cited by 50% of survey respondents, is the “language barrier” between experimentalists and theorists. While structural biology often provides the “what” and computation the “how”, the two frequently operate on disparate spatiotemporal scales. Experimentalists, as noted in our survey, struggle to judge the validity of simulation assumptions (e.g. choice of force field or ensemble), and, accordingly, have difficulties grasping the potential and limitations of a computational experiment. The language barrier also often makes it difficult to translate mathematical abstractions into a concrete biological process, while it may also be difficult for the theorist to fully understand which processes are of true interest to the experimentalist. Another bottleneck is that theorists often lack access to the precise physicochemical metadata required for accurate modeling of wet-lab experiments. To fully capitalize on the potential of integrated computational-experimental scientific approaches, it is important that researchers develop a common language and an ability to provide each other timely feedback, so that they can advance their research in a concerted manner. It is also important that the primary outcome of the simulation can be easily translated into an experimentally measurable parameter. This requires precise theoretical knowledge of the system under investigation, and thus close communication between both parties involved. Addressing this gap requires sustained community effort and the training of a new generation of truly interdisciplinary virologists capable of efficiently bridging the two. Events such as the 2025 EMBO/FEBS Lecture Course on Physical Virology are pivotal in this respect, although their geographic concentration has so far remained problematic. As noted in Section “A 2025 snapshot of the landscape of computational virology”, the Global South is often less present in these meetings and relegated to analytical or simplified computational models due to the “HPC bottleneck”, skewing the global research agenda toward approaches that are “economical” but less detailed (although not necessarily less realistic).

### The “economic” vs. “realistic” paradox: methodological limitations

The second category concerns the biological accuracy of computational models. Because of the vast scale of multicomponent viral systems from the MS2 phage’s 90–dimer lattice to the massive Vault particle (Nuria Verdaguer), researchers face a persistent trade–off. These limitations are deeply intertwined: (i) statistical significance and replicates: many computer simulations suffer from questionable statistical robustness, being biased by starting configurations. Without sufficient replicates, we risk reporting anecdotal molecular events rather than generalizable physical principles. Highly detailed models are so computationally expensive that they rarely explore different experimental conditions (temperature, pH, salt concentration, and mutations); (ii) point–to–point approaches: as highlighted by Rees Garmann and Jodi Hadden–Perilla, the presence of the genome is a major physical driver. Yet, most simulations correspond to virus–like particles rather than infectious virions, due to the lack of high–resolution structures for packaged RNA/DNA. Similarly, the probabilistic nature of glycosylation (the “sugar coat” hijacked from cellular machinery) remains a “dark matter” in computational virology. Current studies rarely address how different viral glycosylation patterns modulate the energy landscape, even though this is crucial for SARS–CoV–2 Omicron binding (as discussed by Conca et al. 2025); (iii) the physicochemical blind spot: a major disconnect persists between simulation parameters and experimental reality. While researchers like Raya Sorkin and Christian Sieben emphasize that pH triggers (e.g. pH 5.0 for fusion) are the “climax” of the viral story, most simulations treat protonation states as static or neutral. Neglecting this pH–dependent mechanism may explain why models fail to capture, e.g. dynamic pore opening in HIV–1 or the pH–dependent vimentin interaction in SARS–CoV–2.

### Newly arising issues

AI tools provide valuable structural templates, yet they struggle to capture the conformational plasticity (e.g. pH–dependent pre– to post–fusion transitions) that dictates viral infection. Their importance is unquestionable, but their use must follow critical criteria and be combined with traditional simulations. Conversely, high–performance simulations generate terabytes of “expensive” data that are rarely shared in formats usable for training physicochemically–aware AI. This lack of FAIR data pipelines represents a missed opportunity for synergy between AI and physics–based modeling.

Beyond accessibility, reproducibility itself is at stake: without open repositories containing full trajectories, parameter files, and physicochemical metadata, many simulations remain anecdotal rather than generalizable. A community–driven initiative equivalent to the PDB but dedicated to simulations could transform this landscape, storing not only final structures but also the underlying dynamics and conditions. Yet, this raises pressing questions of governance and sustainability: who would maintain such servers, how would their costs be covered, and how could equitable access be guaranteed across regions with vastly different economic and HPC infrastructures?

The challenge of equity is particularly acute. Researchers in the Global South already face restricted access to HPC, and without deliberate policies, a “simulation bank” risks reproducing the same asymmetries. Solutions might include regional mirrors, subsidized storage, or compression pipelines to democratize access. Only by ensuring that reproducibility and equity are embedded into the computational ecosystem can the field avoid deepening existing divides.

As Aznar pointed out during her presentation on super–CG models, being “an economic person” is often necessary when navigating the staggering complexity of the viral mesoscale. However, the community’s consensus is that this “economy” must not be a surrogate for physical oversimplification. Bridging the computational–experimental divide requires a new generation of “physicochemically–literate” models—approaches that are both computationally efficient and strictly faithful to experimental conditions (pH, ionic strength, and genomic pressure). Only then will pragmatism and precision advance hand–in–hand to transform the computational microscope from a descriptive tool into a predictive engine for antiviral design.

## Perspectives

The next decade of physical and computational virology will likely focus more intensively on transforming computational models from descriptive tools into predictive engines. Achieving this requires a balanced approach that integrates different simulation scales with physics–aware AI.

Future methodological efforts must better represent the chemical and structural diversity of viral systems. The Martini 3 CG force field remains one of the primary choices for large–scale simulations, particularly for investigating the complex lipid environments of host cell membranes and viral envelopes (Pezeshkian et al. 2023, Pedersen et al. 2025), or for the study of the large conformational changes of viral systems (Cofas-Vargas et al. 2024b, 2026, Olivos-Ramirez et al. 2025, Olivos-Ramirez et al. 2026). Its capacity to handle billion–atom systems is essential for studying the mechanics of viral attachment and entry (Stevens et al. 2023). In parallel, the SIRAH CG framework provides a necessary complementary alternative (Klein et al. 2023). By offering unbiased secondary structure simulations and higher resolution for protein–nucleic acid interactions than traditional CG models, SIRAH is uniquely suited for studying internal virion organization, such as genomic packaging. Rather than relying on a single method, the field is moving toward using these frameworks in combination, to capture the full complexity of the viral replication cycle.

One key objective for future research is to incorporate realistic environmental conditions, pH in particular. Vital biological events, such as endosomal fusion, are triggered by changes in acidity that are often ignored in standard simulations. Constant–pH simulation methods, such as FORTE, can be used to address this limitation, although the full coupling of titration and conformational changes remains a computational open issue (Barroso da Silva et al. 2019). Solving these issues will enable researchers to simulate viruses in their functional environments, providing a more accurate view of pH–dependent phenomena.

AI is expected to transition from predicting static structures to analyzing complex trajectories. The implementation of ML frameworks that respect physical symmetries, such as SO3LR (Kabylda et al. 2025) and other equivariant neural networks, offers a way to achieve high–level precision at a much lower computational cost. When trained on high–quality data, these AI models will be instrumental in identifying rare but functionally critical events, such as pore opening or antibody neutralization escape, which are often difficult to detect in massive datasets.

Finally, ensuring broad access to HPC and promoting the sharing of simulation data (FAIR principles (Amaro et al. 2025)) will be vital for the global advancement of the field and the confidence of experimentalists. As the 2025 EMBO/FEBS Lecture Course on Physical Virology demonstrated, “*all models are bad, but some are useful*”. The goal of the next generation of physical and computational virologists is to make these computer models increasingly useful by grounding them in experimental reality, making them computationally “economic” without sacrificing the fundamental physics of the viral replication cycle. The partnership between experimentalists and computationalists will play a vital role in this process. Training a new generation of researchers proficient in both sides of the same science can also enhance our understanding of viruses. Strategies inspired by Cristina Gomez Navarro are indispensable for addressing current gender bias and fostering a more inclusive and balanced future in physical virology, particularly in computational research.

Despite these challenges, simulations are already delivering valuable insights. As highlighted by our survey, they can anticipate experimental behavior even before wet–lab data exist, guiding hypotheses and reducing trial–and–error by ensuring targeted experimental designs, thereby saving time and resources. This predictive capacity demonstrates that, when computation and experimentation advance together, the field has the potential not only to unravel the hidden world of viruses but also to translate this knowledge into tangible solutions, ultimately improving human health and quality of life. Areas of application are many; they range from accurately predicting viral evolution, e.g. in the context of vaccine development, to accelerating antiviral drug development by facilitating the design of optimal interaction partners. They also promise to broaden our fundamental understanding of the mechanisms of viral infection by providing unprecedentedly accurate descriptions of the underlying biological processes relying on physical and chemical principles.

## Supplementary Material

fuag050_Supplemental_File

## Data Availability

The data underlying this article are available in the article and in its online supplementary material. Individual survey responses cannot be shared publicly to protect the privacy of the participants.
