# Supplementary material for: Computational methods in physical virology: a critical perspective across lengths and timescales
Source: FEMS Microbiol Rev. 2026 Sep 22;50:fuag050. doi: 10.1093/femsre/fuag050 (PMC13637490; doi:10.1093/femsre/fuag050)
Supplement: Supplementary file 1 [file fuag050_supplemental_file.docx]

**Supplementary Information**

**Computational Methods in Physical Virology: A Critical Perspective across lengths and timescales**

Adolfo Poma*¹, Luis F. Cofas-Vargas², Fernando L. Barroso da Silva*^3,4^, Sergio Pantano*^5,6^, Marta Bally*^7^

¹Department of Biosystems and Soft Matter, Institute of Fundamental Technological Research, Polish Academy of Sciences, ul. Pawinskiego 5B, 02-106, Warsaw, Poland

²Departamento de Química, Universidad Autónoma Metropolitana-Iztapalapa, Ciudad de México, México

^3^Department of Biomolecular Sciences, School of Pharmaceutical Sciences at Ribeirão Preto, University of São Paulo, Ribeirão Preto, São Paulo, Brazil

^4^Department of Chemical and Biomolecular Engineering, North Carolina State University, Raleigh, NC, USA

^5^Institut Pasteur de Montevideo, Montevideo, Uruguay;

^6^Área Bioinformática, Departamento DETEMA, Facultad de Química, Universidad de la República, Montevideo, Uruguay

^7^Department of Clinical Microbiology, Wallenberg Centre for Molecular Medicine, Umeå Centre for Microbial Research & and Science for Life Laboratory, Umeå University, Umeå, Sweden

Oral and Poster presentations (**bold** indicates the main speaker or poster presenter)

1) Structural Dynamics of Multiprotein Complexes: Viral assemblies and the Vault Particle, **Nuria Verdaguer**, Molecular Biology Institute of Barcelona, IBMB-CSIC
2) Packaging and discovering RNA bacteriophage genomes, **Rees Garmann**,
   Department of Chemistry and Biochemistry, San Diego State University
3) Learning the RNA Jigsaw Rules from Viruses with Multi-segmented Genomes **Alexander Borodavka,** University of Cambridge
4) Science as Service: how to use storytelling to bridge the gap between your science and community impact, **Heather Masson-Forsythe*** Science Policy and Communication
5) Combining cryo electron microscopy and mass spectrometry to study the polyclonal antiviral antibody response, **Joost Snijder**, Utrecht University
6) Icosahedral virus capsids through the computational microscope **Jodi Hadden-Perilla**, University of Delaware
7) Dynamic nanomachines: Unravelling a virus maturation pathway through cryo-EM, **Roger Castells-Graells**, Spanish National Cancer Research Centre (CNIO) and Department of Biochemistry and Metabolism, John Innes Centre
8) Structural diversity of archaeal viruses **Mart Krupovic**, Institut Pasteur
9) More than simple adhesion: Interactions between glycans and small DNA tumor viruses for cell entry, **Mario Schelhaas**, Institute of Cellular Virology, University of Münster, Germany
10) New Tools and Mechanistic Insights into Viral Fusion, **Raya Sorkin,** School of Chemistry, Faculty of Exact Sciences, Tel Aviv University, Tel Aviv 6997801, Israel.
11) Speak friend and enter – deconstructing the virus-cell infection synapse, **Christian Sieben**, Nanoscale Infection Biology Group, Helmholtz Centre for Infection Research (HZI), Braunschweig, Germany
12) “Locking on” to the early stages of the virus-cell interaction with three-dimensional active- feedback microscopy, **Kevin Welsher,** Duke University
13) 4D single particle microscopy and AI Synergy: A New Era in Studying Virus Entry Mechanisms **Nikos Hatzakis,** University of Copenhagen, Department of Chemistry
14) Understanding and Breaking Barriers for Women in Science **Cristina Gomez-Navarro**,
    Universidad Autónoma de Madrid
15) High-fidelity in vitro assembly and cargo packaging in encapsulin protein cages **Yu Heng Lau**, School of Chemistry, The University of Sydney
16) Cell-free synthesis of viral membrane proteins for vaccine nanocarriers, **Neha Kamat,**
    Northwestern University
17) Virus-like particles at the interface of soft materials, **Frank Sainsbury**, Institute for Biomedicine and Glycomics, Griffith University, Australia
18) Funcionalized viral protein assemblies as scaffolds for neural tissue engineering, **Laura A. Palomares**, Instituto de Biotecnologia. Universidad Nacional Autónoma de México
19) Dissection of enveloped virus membrane fusion mechanisms using cell-free assays **Enya Qing**, Tom Gallagher, Loyola University Chicago Stritch School of Medicine
20) Conducting polymer devices to study virus host interactions, **Roisin M. Owen**,
    University of Cambridge
21) Chemical interactions of empty/full AAV capsids measured with single particle AFM **Caryn Heldt**, Michigan Technological University

Posters (**bold** indicates the main presenter)

1) Investigation of the designed protein cages polymorphism by cryoEM, **Artur Biela**, NSRC SOLARIS, Jagiellonian University
2) The assembly of Chikungunya core is driven by highly conserved electrostatic interactions, **Mauricio Comas-Garcia,** aDepartment of Sciences, Autonomous University of San Luis Potosi, Mexico and Research Center for Health Sciences and Biomedicine, Autonomous University of San Luis Potosi, Mexico
3) New insights into tobacco mosaic virus: stability, disassembly and uncoating, **Alejandro Díez Martínez**, Universidad Autónoma de Madrid
4) Cryo-EM Insights into Intermediate Structures of BMV-Derived VLPs, **Anna Urbanowicz,** Institute of Bioorganic Chemistry, Polish Academy of Sciences, Poznań, Poland
5) Exploring the mysteries surrounding the formation of RCNMV virus-like particles, **Martyna Kordyś**,, Laboratory of Protein Engineering, Institute of Bioorganic Chemistry, Polish Academy of Sciences, Poznań, Poland
6) Exploring the stability and conformational dynamics of SARS-CoV-2 spike protein variants and their complexes with a potent nanobody, **Adolfo Poma Bernaola,** Department of Biosystems and Soft Matter, Institute of Fundamental Technological Research, Polish Academy of Sciences, Warsaw, Poland
7) Antigen Display on VLP’s: A Paradigm Shift in COVID-19 Vaccine Design and Immunization Strategies, **Paulomi Goswami**, Indian Institute of Technology, Delhi
8) Membrane composition asymmetry is a defense mechanism against viral infection, **Gonen Golani,** Department of Physics, University of Haifa
9) Drosophila C Virus and Drosophila melanogaster: A Model for Studying Wolbachia-Induced Antiviral Protection, **Yuliya Chykunova**, Molecular Mechanisms of Symbiosis Laboratory, Institute of Environmental Sciences, Faculty of Biology, Jagiellonian University, Cracow, Poland
10) Snapshot of adenovirus disassembly reveals a portal-like structure and reorganization of internal capsid and core proteins, **Marta Martínez**, Department of Macromolecular Structures, Centro Nacional de Biotecnología (CNB-CSIC), Madrid, Spain
11) Dynamic assembly of non-quasi-equivalent, pentamer-based protein cages and nanotubes, **Yusuke Azuma**, Jagiellonian University
12) Understanding rabies virus using whole genome sequence data, **Ankeet Kumar,** Indian Institute of Science, Bangalore
13) Multiple-enzyme kinetics in single monodisperse nanoreactors unveil correlations between broadly distributed catalytic rates, **Klara Strobl,** Universidad Autónoma de Madrid, Spain
14) Viral Genome Debundling and Karyopherin Interplay during Influenza A Virus Cellular Entry, **Takuto Washizawa,** Nagoya University School of Medicine and ETH Zurich, Institute of Pharmaceutical Sciences
15) Energetics and Mechanisms of Membrane Remodelling in SARS-CoV-2 Assembly, **Nils Oliver Winkler,** Institute for Theoretical Physics and Bioquant, Heidelberg University
16) Developing modular virus-like particles as vaccine candidate for feline coronavirus, **Antonina Naskalska,** Malopolska Centre of Biotechnology, Jagiellonian University, Krakow, Poland
17) Integrative structural biology of A T=219 marine giant virus, **Carmen San Martín**, Centro Nacional de Biotecnología (CNB-CSIC), Spain
18) Models for RNA folding within virions of the Zika virus, **Yavor Novev,** Institute of Genetics and Cancer, University of Edinburgh
19) In vitro reconstituted, antibody-conjugated VLPs as a platform for targeted in vivo delivery of self-amplifying mRNA genes, **Ana Luisa Duran Meza**, University of California Los Angeles
20) Structural and functional analysis of UL12.5 – a HSV-1 DNAse involved in reactivation of viral replication and Alzheimer’s disease progression, **Xiaoyu Wan**, Department of Molecular Pharmacology, Groningen Research Institute of Pharmacy (GRIP), University of Groningen, The Netherlands
21) Revealing the mechanisms of virus assembly with single-particle kinetics, **Pedro Buzón,** Biochemistry Institute, University of Zurich, Zurich 8057, Switzerland
22) Revealing SARS-CoV-2 replication transcription super-complex (RTC)architecture and dynamics via native MS, **Fatema-Aqila Said,** CSSB Centre for Structural Systems Biology, Deutsches Elektronen-Synchrotron DESY & Leibniz Institute of Virology (LIV) & University of Lübeck, Notkestraße 85, 22607 Hamburg, Germany and Institute of Chemistry and Metabolomics, University of Lübeck, Ratzeburger Allee 160, 23562 Lübeck, Germany
23) Magic sizes enable minimal-complexity, high-fidelity assembly of programmable shells, **Botond Tyukodi,** Babes-Bolyai University, Department of Physics, Centre International de Formation et de Recherche Avancées en Physique, 077125 Bucharest- Magurele, Romania and Martin Fisher School of Physics, Brandeis University, Waltham, Massachusetts 02454, USA
24) The metabolite IP6 modulates the energy landscape and promotes pore opening in the protein lattice of the HIV-1 capsid, **Alejandro Valbuena**, Centro de Biología Molecular Severo Ochoa (CSIC-UAM), Campus of the Universidad Autónoma de Madrid, 28049 Madrid, Spain
25) Insights into the mode of action of SARS-CoV-2 putative fusion peptides and their potential as antiviral targets, **Carolina C. Buga,** Instituto de Tecnologia Química e Biológica António Xavier, Universidade Nova de Lisboa, Oeiras, Portugal, Faculdade de Medicina, Universidade de Lisboa, Lisboa, Portugal and Gulbenkian Institute for Molecular Medicine, Lisboa, Portugal
26) Ultrapotent SARS coronavirus-neutralizing single-domain antibodies that clamp the spike at its base, **Sieglinde De Cae,** VIB Center for Medical Biotechnology and Department of Biochemistry and Microbiology, Ghent University
27) Protein and Nucleic Acids interaction for Viral Nucleocapsid Assembly, **Ruana Cardoso-Lima,** Department of Physics and Astronomy, Vrije Universiteit Amsterdam,, Amsterdam, The Netherlands
28) Mayaro virus: filling gaps in understanding biological physics **Joel Félix Silva Diniz Filho**, Biophysics and Nanossystems Laboratory, Department of Physics, Federal University of Maranhão, São Luís, Maranhão, Brazil and Brazilian Biosciences National Laboratory (LNBio), Brazilian Center for Research in Energy andMaterials (CNPEM), Campinas, Brazil
29) The cellular thermal proteome during semliki forest virus infection, **David Mentrup,** Karolinska Institutet, MTC
30) Assembled genome structure helps influenza A virus uncoating, **Yasuyuki Miyake,** Nagoya University Graduate School of Medicine and Nagoya University, Institute for Advanced Research (IAR)
31) In cellulo synthesis of virus-like particles containing specific self-amplifying RNA, **David Silverio Moreno Gutierrez,** Department of Chemistry and Biochemistry, University of California Los Angeles
32) Molecular insights into SARS-CoV-2 N-protein interactome by native mass spectrometry, **Jonas Schröder,** CSSB Centre for Structural Systems Biology, Deutsches Elektronen-Synchrotron DESY & Leibniz Institute of Virology (LIV) & University of Lübeck, Notkestraße 85, 22607 Hamburg, Germany and Institute of Chemistry and Metabolomics, University of Lübeck, Ratzeburger Allee 160, 23562 Lübeck, Germany
33) Viral particles studied by High-Speed AFM, **Ján Sabó,** Moleculaire Biofysica, Zernike Institute for Advanced Materials, Rijksuniversiteit Groningen
34) Interplay of glycans and adsorption-driven deformation in SARS-CoV-2 RBD variants onto surfaces with different hydrophobicity, **Antonio Miguel Bosch Fernandez,** Departamento de Física Teórica de la Materia Condensada, Universidad Autónoma de Madrid, Madrid, Spain
35) Investigating glycan-mediated norovirus attachment to host plasma membranes using enzymatic digestion of receptor structures, **Hudson Pace**, Department of Clinical Microbiology and Wallenberg Centre for Molecular Medicine, Umeå, University, Umeå, Sweden
36) Exploring the Biophysical Properties of Melbournevirus Using Atomic Force Microscopy, **Adrián Cámara**, Departamento de Física de la Materia Condensada, Universidad Autónoma de Madrid, Spain
37) Quantitative electrostatics for tracing icosahedral virus capsids adsorption onto flat substrates, **Willy Menacho,** Department of Mechanical Engineering, Universidad Tecnica Federico Santa María, 2390123 Valparaíso, Chile
38) Synthesis, Assembly and Characterization of Plasmonic Viromimetic Nanoparticles, **Maria-Lorena Jinga,** International Center for Advanced Training and Research in Physics (CIFRA), Magurele, Romania, National Institute of Materials Physics (NIMP), Magurele, 077125, Romania and University of Bucharest, Doctoral School of Physics, Magurele, Romania
39) Sulfation patterns of heparan sulfate influence the physicochemical characteristics of papillomavirus interactions for entry, **Fouzia Bano,** Department of Clinical Microbiology and Wallenberg Centre for Molecular Medicine, Umeå University, Umeå, Sweden
40) Molecular mechanisms behind filovirus entry: exploring cell attachment and entry potential of different filovirus-encoded glycoproteins, **Malgorzata Grau,** Department of Clinical Microbiology, Umeå University, Umeå Sweden, Wallenberg Centre for Molecular Medicine at Umeå University and Umeå Centre for Microbial Research (UCMR), Umeå University, Sweden
41) Direct glycoproteomics by native MS from intact Influenza virions, **Marius Lauffer,** Leibniz Institute of Virology, Martinistraße 52, 20251 Hamburg, Germany, CSSB Centre for Structural Systems Biology, Deutsches Elektronen-Synchrotron DESY & Leibniz Institute of Virology (LIV) & University of Lübeck, Notkestraße 85, 22607 Hamburg, Germany and Institute of Chemistry and Metabolomics, University of Lübeck, Ratzeburger Allee 160, 23562 Lübeck, Germany
42) Three-dimensional structure of bacteriophage Nocturne116, **Jānis Rūmnieks**, Latvian Biomedical Research and Study Center, Rātsupītes 1, Riga 1067, Latvia
43) Nipah virus attachment and fusion glycoproteins regulate distinct facets of virus-host fusion, **Susan Daniel**, Cornell University
44) Interfacial Partitioning of Conserved Tyrosine Y819 Optimally Positions the SARS-CoV-1 Fusion Peptide during Host Cell Membrane Interactions and Entry, **Ambika Pachaury,** Department of Biomedical Engineering, Boston University
45) An assembly-line mechanism for in-vitro encapsulation of fragmented cargo in virus-like particles, **Ayesha Amjad,** Department of Chemistry, Indiana University, Bloomington, IN 47405, United States
46) Molecular simulations of nucleic acid packaging in viruses, **Kush Coshic,** Max Planck Institute of Biophysics, Frankfurt, Germany and University of Illinois at Urbana-Champaign, IL, USA
47) Virus 14С-labeling for ultrasensitive quantification by accelerator mass-spectrometry, **Elena Prokopyeva,** Faculty of Fundamental Medicine, Novosibirsk State University, Novosibirsk, Russia
48) EfDRV as a model for exploring immature deltaretroviral assembly, **Darya Chernikova,** Institute of Science and Technology Austria, Klosterneuburg, Austria
49) RNA polarization under icosahedral confinement **Horacio V. Guzman**, Instituto de Ciencia de Materiales de Barcelona, CSIC
50) The role of electrostatic interactions between the E1 and E2 proteins of the Chikungunya virus in viral assembly and entry, **Andrea Castorena**, Universidad Autónoma de San Luis Potosí
51) Structure of a thermostable phage’s portal vertex and neck complex illuminates its maturation process, **Brian Kelch,** University of Massachusetts Chan Medical School
52) Next-generation adjuvant systems containing furfuran drive potent adaptive immunity and host defense as a viral vaccine adjuvant, **Hyeong Won Kim,** Centre for Foot-and-Mouth Disease Vaccine Research, Animal and Plant Quarantine Agency, Gimcheon-si, Gyeongsangbuk-do, Republic of Korea
53) Small molecule, as a viral vaccine adjuvant, induces robust host defense through the modulation of innate and adaptive immune response, **Min Ja Lee,** Animal and Plant Quarantine Agency
54) Structural and functional features of a conserved DNA-binding domain in herpesviral proteins as a basis for innovative targeted therapies, **Fatama A. Sornaly,** University of Lübeck
55) Investigating viral membrane fusion with a controlled giant plasma membrane vesicles based platform, **Inbar Yosibash,** School of Chemistry, Tel Aviv University, Israel
56) Unveiling the role of the capsid template in superradiant virus-like particles **Kristen White**, Indiana University, Department of Chemistry
57) Engineered mini FnCas9 variants for efficient in-vivo therapeutic genome editing via Virus- Like Particles (VLPs), **Sajal Sarkar,** CSIR-Institute of Genomics & Integrative Biology, Mathura Road, New Delhi, 110025, India, Academy of Scientific & Innovative Research (AcSIR), Ghaziabad, 201002, India and GROW Research Laboratory, Narayana Nethralaya Foundation, Bangalore, Karnataka,560099, India
58) Room-temperature superradiance from a virus supported multi-chromophore antenna **Irina Tsvetkova**, Chemistry department, Indiana University, Bloomington, IN, USA
59) Fabrication and characterization of microfluidic channels for investigating protein- nanoparticle interactions within viromimetic assemblies, **Daciana Botta,** National Institute of Material Physics, Atomistilor 405A, Magurele, Romania and International Center for Advanced Training and Research in Physics (CIFRA), Magurele, Romania
60) Direct observation of filovirus interaction with the endothelial glycocalyx, **Dario Valter Conca,** Department of Clinical Microbiology, Umeå University, Umeå 901 87, Sweden, Wallenberg Centre for Molecular Medicine (WCMM), Umeå University, Umeå 901 87, Sweden and Umeå Centre for Microbial Research (UCMR), Umeå University, Umeå 901 87, Sweden
61) Precise quantification of cell-to-cell fusion in culture by deep learning **Suman Khan**, Department of Biomolecular Sciences, Weizmann Institute of Science, Rehovot 7610001, Israel
62) Enhanced nucleic acid packaging, stability, and delivery efficiency via incorporation of the HIV-1 NC domain in self-assembling protein nanoparticles, **Ximena Del Toro Ríos**, Instituto de Química, Universidad Nacional Autónoma de México
63) Single-particle cryo-EM reveals a novel form of viral 3D tRNA mimicry, **Steve Bonilla**,
    Laboratory of RNA Structural Biology and Biophysics, The Rockefeller University
64) Sars_CoV_2_dynamics, **Aleksandar Atemin**, Institute of Molecular Biology, Bulgarian Academy of Sciences, Sofia, Bulgaria
65) Chemical strategies for protein cargo packaging and display on Virus-Like Particles **Tariq Hussain**, Department of Molecular and Cellular Biochemistry, Indiana University, Bloomington, IN, USA
66) Cell free synthesis of vaccinia non-structural regulatory proteins to investigate their host membrane remodeling activities during morphogenesis, **Juliana Carten**, Robert Frederick Smith School of Chemical & Biomolecular Bioengineering, Cornell University, Ithaca NY 14853
67) Physical virology in virus manufacturing **Claudia Simon**, Boehringer Ingelheim, Viral Therapeutics Centre
68) Using cell-free assembled virus-like proteoliposome complexes to deliver cargo directly to cellular cytosol, **Ekaterina Selivanovitch**, Cornell University
69) Developing virus-like particles for Equine rotavirus vaccine candidates, **ThanhTam Pham**, Jeju National University
70) Utilizing Multi-Parametric Surface Plasmon Resonance (MP-SPR) and Quartz Crystal Microbalance with Dissipation Monitoring (QCMD) for real-time analysis of virus-peptide interactions, **Justas Svirelis**, BioNavis Ltd., Tampere, Finland
71) Structure and mechanism of the SARS-CoV-2 methylation complex **Alex Matsuda**, Virogenetics Laboratory of Virology, Malopolska Centre of Biotechnology, Jagiellonian University, Kraków, Poland and Doctoral School of Exact and Natural Sciences, Jagiellonian University, Kraków, Poland
72) Structural studies of coxsackievirus B1 virus-like particles, **Alesia Levanova,** University of Helsinki, Finland
73) From validation to prediction: A biophysical tool for mapping SARS-CoV-2 variant evolution, **Fernando Luis Barroso da Silva**, School of Pharmaceutical Sciences at Ribeirao Preto/University of Sao Paulo
74) Bacteriophage T7: link between a virtual and an experimental, Atomic Force Microscope (AFM), **Maria Aznar Palenzuela,** Dept. de Física Fonamental, Universitat de Barcelona, Spain
75) Towards Tunable Meta-Stability in De Novo Protein Assemblies, **Miguel Atienza Juanatey**, Alena Khmelinskaia, aLMU-Munich
76) Modeling the Cirocvirus genome reveals clues on DNA-protein packing and suggests conformational quasispecies, **Sergio Pantano,** Institut Pasteur de Montevideo
77) Mammalian cell platform for porcine circovirus 2: A new thermostable vaccine prototype, **Claudia Ortega**, Institut Pasteur de Montevideo
78) Examining virus-cell interactions via confocal iSCAT microscopy, **David Albrecht,** Max Planck Institute for the Science of Light and Max-Planck-Zentrum für Physik und Medizin cFriedrich Alexander University
79) Biophysical mechanisms of lipid nanoparticle-mediated RNA delivery: Interfacial interactions of lipid nanoparticles with supported lipid bilayers, **Simon Niederkofler,** Chalmers University of Technology
80) Generation of a one-plasmid Alphavirus replicon/helper system, **Mauricio Comas-Garcia,** Research Centre for Health Sciences and Biomedicine, Autonomous University of San Luis Potosi and Department of Sciences, Autonomous University of San Luis Potosi
